# Sequential Pressurized and Supercritical Extraction Strategies for the Recovery of Phenolic Compounds and Grape Bagasse Valorization

**DOI:** 10.3390/molecules31132314

**Published:** 2026-07-01

**Authors:** Vanessa Souza Carvalho, Jonas da Silva, Lucas Cantão Freitas, Sandra Regina Salvador Ferreira, Marcos Lúcio Corazza

**Affiliations:** 1 Department of Chemical Engineering, Federal University of Paraná, Curitiba 81531-990, PR, Brazil; vanessasouza@ufpr.br (V.S.C.); lucas.cantao@ufpr.br (L.C.F.); 2 Department of Chemical Engineering and Food Engineering, Federal University of Santa Catarina, Florianópolis 88040-900, SC, Brazil; jonasengalimentos@gmail.com (J.d.S.); s.ferreira@ufsc.br (S.R.S.F.); 3 Federal Institute of Paraná, Colombo 83403-515, PR, Brazil

**Keywords:** grape bagasse, phenolic compounds, pressurized liquid extraction, subcritical water extraction, sequential extraction

## Abstract

Grape bagasse is an abundant agro-industrial by-product and an important source of phenolic compounds with antioxidant properties. This study evaluated Soxhlet extraction, supercritical fluid extraction (SFE), pressurized liquid extraction (PLE), subcritical water extraction (SWE), and sequential extraction strategies for recovering bioactive compounds from grape bagasse. Box–Behnken designs were applied to SFE and PLE to evaluate process effects on extraction yield, while total phenolic content (TPC), total anthocyanin content (TAC), and antioxidant activity (ABTS) were additionally determined for PLE extracts. Hydroethanolic extractions showed greater selectivity toward phenolic compounds, whereas water-based extractions promoted higher yields associated with additional polar constituents. In SWE, increasing temperature enhanced extraction yield and phenolic recovery, although anthocyanin contents decreased under more severe thermal conditions. SWE provided higher extraction yields than PLE with comparable phenolic content and antioxidant activity, suggesting the recovery of additional highly polar non-phenolic compounds, whereas PLE resulted in higher extraction yields than SFE. Sequential extraction demonstrated that the first step accounted for most of the phenolic recovery and antioxidant activity, while the second aqueous step increased overall extraction yield. The sequential PLE–SWE route resulted in the highest TPC (198.0 mg GAE g^−1^) and antioxidant activity (2321 μmol TE g^−1^), demonstrating the potential of sequential extraction for grape bagasse fractionation and valorization.

## 1. Introduction

Grape processing generates large amounts of solid residues, commonly referred to as grape bagasse or pomace, which consist mainly of skins, seeds, and residual pulp. These by-products represent an important environmental challenge for the wine and juice industries, but also a valuable source of bioactive compounds, particularly phenolic compounds with recognized antioxidant properties [1,2,3,4,5].

Phenolic compounds present in grape bagasse, including flavonoids, phenolic acids, and anthocyanins, have attracted increasing attention due to their potential applications in food, pharmaceutical, and cosmetic products [2,5,6]. However, their recovery is strongly influenced by the extraction technique and process conditions, as these compounds are often associated with complex lignocellulosic matrices that can limit solvent accessibility and mass transfer [7,8]. Previous studies have demonstrated that the extraction efficiency and selectivity of grape bagasse phenolics are highly dependent on solvent polarity, extraction temperature, and method [9,10]. Hydroethanolic mixtures are frequently reported as suitable solvents for recovery phenolic compounds from grape pomace, providing a balance between extraction yield and phenolic selectivity, while water-based systems often promote the co-extraction of additional polar constituents. In addition, considerable variability in extraction yield, total phenolic content, and antioxidant activity has been reported in the literature, depending on the operational conditions and extraction technologies employed [10,11,12].

Conventional extraction methods, such as Soxhlet extraction, are widely used as reference techniques due to their simplicity and reproducibility. Nevertheless, they are associated with long extraction times, high solvent consumption, and low selectivity [13]. In recent years, alternative extraction technologies have been increasingly explored to overcome these limitations. Supercritical fluid extraction (SFE), particularly using CO_2_, is considered an environmentally friendly technique due to its low toxicity and easy solvent removal; however, its ability to extract polar compounds is limited, often requiring the use of co-solvents to improve efficiency [14,15]. For grape pomace applications, SFE has shown potential for the recovery of specific bioactive compounds, although extraction yields and phenolic recovery are frequently lower than those obtained using hydroethanolic pressurized systems due to the limited polarity of supercritical CO_2_ [16,17].

Pressurized liquid extraction (PLE) has emerged as an effective alternative for the extraction of bioactive compounds from plant matrices. By operating at elevated temperatures and pressures, PLE enhances solvent penetration, reduces viscosity, and improves mass transfer, leading to higher extraction efficiencies, particularly for polar compounds when using solvents such as ethanol or ethanol–water mixtures [18,19]. Several studies involving grape bagasse have reported high phenolic recoveries and antioxidant activities using PLE under hydroethanolic conditions, highlighting the influence of temperature, solvent composition, and liquid-to-solid ratio on extraction performance [10,12,20]. Subcritical water extraction (SWE) has also gained increasing attention as a green extraction technique. At elevated temperatures, water exhibits decreased dielectric constant and increased diffusivity, allowing to solubilize a broader range of compounds, including moderately polar substances, without the need for organic solvents [21,22]. However, the use of highly polar solvents or high temperatures may reduce extraction selectivity, promoting the co-extraction of non-target compounds such as sugars and other low-molecular-weight constituents [23]. For grape pomace, SWE has been associated with high extraction yields and expressive antioxidant activity, although elevated temperatures may also promote the extraction of non-phenolic compounds and the degradation of thermolabile constituents such as anthocyanins [24,25].

In this context, sequential extraction strategies have been proposed as a way to improve both extraction efficiency and selectivity by combining solvents or techniques with different properties. This approach enables the fractionation of complex matrices based on compound polarity, allowing the recovery of different classes of compounds in separate steps [26,27,28]. Despite the growing body of literature on grape pomace extraction, studies providing a systematic comparison of conventional, supercritical, and pressurized extraction technologies, as well as their integration into sequential fractionation strategies, remain scarce. Consequently, the influence of extraction route on compound selectivity, phenolic recovery, and biomass valorization has not yet been fully elucidated.

Therefore, the aim of this study was to evaluate and compare different extraction techniques, including Soxhlet, SFE with co-extractant, PLE, and SWE, for the recovery of phenolic compounds from grape bagasse. Additionally, sequential extraction strategies combining these techniques were investigated to assess their potential for improving extraction yield and selectivity. This integrated approach enables the assessment of extraction performance, selectivity, and biomass fractionation across conventional, supercritical, and pressurized technologies, providing insights into their application for grape bagasse valorization within a biorefinery framework.

## 2. Results

### 2.1. Conventional Extraction (Soxhlet) as Baseline

Soxhlet extraction was performed using solvents with different polarities (ethanol 100%, 70%, 50%, and water) to establish a baseline for comparison with intensified extraction techniques. The extraction yield and composition of the extracts were strongly influenced by solvent polarity.

As shown in Table 1, the highest extraction yield was obtained using water (26.1%), followed by hydroethanolic mixtures (17.5–17.8%) and absolute ethanol (14.0%). Despite the higher yield, aqueous extraction resulted in relatively low total phenolic content (TPC = 54.6 mg GAE g^−1^), suggesting the co-extraction of non-phenolic compounds, such as soluble sugars.

In contrast, hydroethanolic mixtures (50–70% ethanol) provided significantly higher TPC values (98.2–100.3 mg GAE g^−1^), indicating that solvents of intermediate polarity enhance the selective recovery of phenolic compounds. A similar trend was observed for antioxidant activity (ABTS), with the highest values obtained for 50% and 70% ethanol.

Total anthocyanin content (TAC) was also influenced by solvent composition, with the highest value observed for 70% ethanol (11.5 mg CY g^−1^), while water extraction resulted in substantially lower values.

These results demonstrate that higher extraction yields do not necessarily correspond to higher selectivity for phenolic compounds. Therefore, solvent polarity plays a key role not only in extraction efficiency but also in extract composition, providing an important reference for evaluating intensified extraction techniques.

### 2.2. Effects of Process Variables on SFE and PLE (Box–Behnken Design)

**Figure 1:**
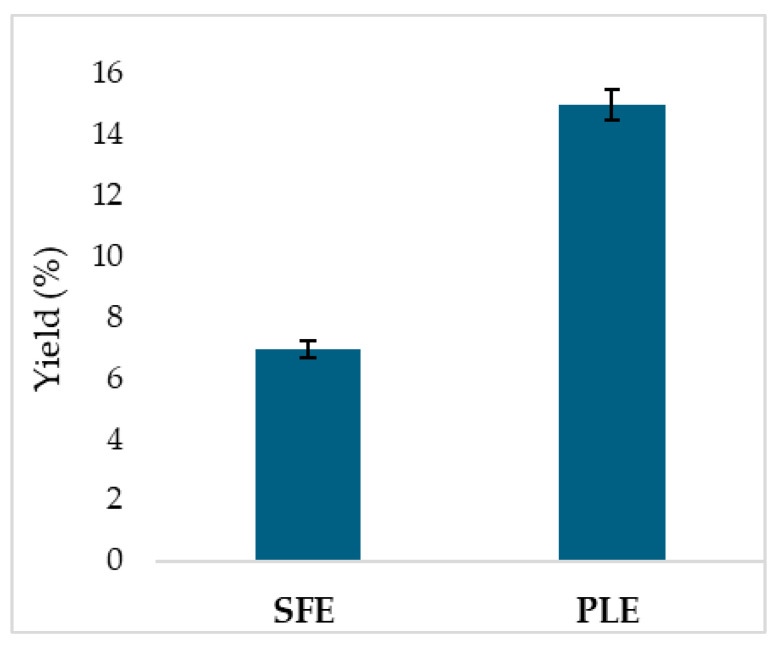
Extraction yield obtained from the triplicate central-point experiments of the Box–Behnken design (BBD) for supercritical fluid extraction (SFE) and pressurized liquid extraction (PLE). Error bars represent the standard deviation (n = 3). This behavior is consistent with the results obtained from Soxhlet extraction, where solvents of intermediate polarity enhanced the recovery of extractable compounds, suggesting that PLE, using ethanol as solvent, provides a more suitable environment for the extraction of polar constituents from grape bagasse.

To provide an initial comparison between the extraction techniques, the yields obtained at the central experimental conditions of SFE and PLE are presented in Figure 1. As observed, PLE resulted in substantially higher extraction yields (~15%) compared to SFE (~7%), considering the central points of the Box–Behnken designs adopted for each extraction method, indicating greater extraction efficiency.

As PLE resulted in substantially higher extraction yields than SFE, chemical characterization was performed only for extracts obtained by this method. It should be noted that lower extraction yields do not necessarily imply lower concentrations of phenolic compounds in the obtained extracts. However, under the evaluated conditions, SFE provided considerably lower extract recoveries than PLE, indicating a lower overall recovery of grape bagasse phenolics. In addition, the reduced extract amounts obtained by SFE limited further chemical characterization within the scope of this study. Therefore, total phenolic content (TPC), total anthocyanin content (TAC), and antioxidant activity (ABTS) were evaluated only for PLE extracts, while SFE was assessed based on extraction yield.

The fitted regression models and statistical parameters for SFE and PLE are summarized in Table 2. All models presented satisfactory coefficients of determination (R^2^ = 0.88–0.94) and non-significant lack of fit (*p* > 0.05), indicating adequate representation of the experimental data.

The complete experimental design and corresponding response values are provided in the Supplementary Materials (Tables S1 and S2), together with the complete ANOVA tables for all fitted models (Tables S3–S7).

For SFE, extraction yield was significantly influenced by temperature and ethanol-to-solid ratio. These variables affected the extraction performance; however, the overall yields remained lower compared to PLE, reflecting the limited capacity of supercritical CO_2_ to solubilize highly polar compounds.

For PLE, the evaluated variables showed distinct effects depending on the response. Extraction yield was mainly influenced by temperature, with higher temperatures promoting increased extraction yields. A similar effect was observed for antioxidant activity (ABTS), indicating that temperature also plays an important role in enhancing the extraction of antioxidant compounds. In contrast, temperature did not show a significant effect on TPC and TAC, indicating that phenolic recovery was more dependent on solvent composition and interactions between variables. Extraction yield ranged from 11.7 to 19.0%, with the highest value obtained at 80 °C, 70% ethanol, and a liquid-to-solid ratio of 33.33 mL/g. Total phenolic content varied between 32.1 and 129.0 mg GAE g^−1^, while TAC ranged from 3.2 to 25.8 mg CY g^−1^. Antioxidant activity (ABTS) values ranged from 324 to 1556 μmol TE g^−1^.

**Figure 2:**
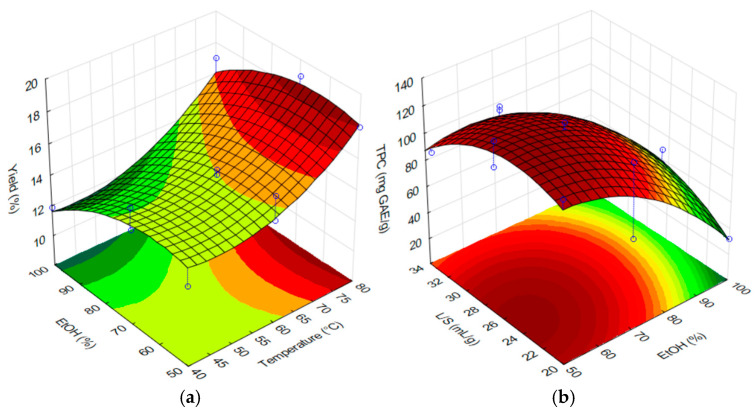
Response surface plots obtained for pressurized liquid extraction (PLE): (**a**) extraction yield (%) as a function of temperature and ethanol concentration, and (**b**) total phenolic content (TPC, mg GAE g^−1^) as a function of ethanol concentration and liquid-to-solid ratio. The colored surfaces represent the response predicted by the fitted quadratic model, while the blue circles indicate the experimental design points.

Response surface plots were constructed for extraction yield and TPC in PLE (Figure 2). The results confirm that temperature is a key factor for increasing extraction yield, whereas TPC was primarily influenced by ethanol concentration and liquid-to-solid ratio, both showing significant quadratic effects.

Additional response surface plots for all evaluated responses and variable combinations are presented in the Supplementary Materials (Figures S2–S6).

The response surface plots demonstrate the importance of properly selecting extraction conditions, since significant variations in extraction yield and phenolic recovery were observed depending on the combination of process variables. In particular, the presence of quadratic effects and curved response surfaces indicates that extreme conditions do not necessarily maximize phenolic recovery, reinforcing the relevance of process optimization for balancing extraction yield and selectivity.

### 2.3. Subcritical Water Extraction

Subcritical water extraction (SWE) was evaluated at 80, 100, and 120 °C, under fixed pressure and liquid-to-solid ratio conditions corresponding to the central values used in PLE. The extraction yield increased with temperature, with the highest value obtained at 120 °C (Table 3).

Increasing SWE temperature promoted higher extraction yields, TPC, and antioxidant activity. However, the increase in extraction yield was more pronounced than that observed for PLE (26.7% versus 19.0%, respectively), while comparable TPC and antioxidant activity values were maintained. These results suggest the co-extraction of additional highly polar compounds under SWE conditions. In contrast, anthocyanin contents decreased at higher temperatures, indicating the thermal sensitivity of these compounds.

Based on these results, SWE at 120 °C was selected for the sequential extraction approach, aiming to recover remaining polar compounds after the initial extraction step.

### 2.4. Performance of Sequential Extraction Strategies

Sequential extraction strategies were evaluated to explore the potential of combining extraction techniques with different selectivity. Two approaches were investigated: a low-pressure (↓P) sequence based on Soxhlet extraction with solvents of increasing polarity, and a high-pressure (↑P) sequence combining PLE and SWE (Table 4).

In both cases, the first extraction step accounted for most of the phenolic compounds and antioxidant activity, whereas the second step mainly increased the overall extraction yield, with limited changes in TPC and antioxidant activity and only negligible anthocyanin recovery, suggesting that water extraction under Soxhlet or subcritical conditions may have promoted the recovery of highly polar non-phenolic compounds.

Although the low-pressure sequential route resulted in higher overall extraction yield, the high-pressure route provided higher overall TPC, TAC, and antioxidant activity, indicating greater selectivity toward phenolic compounds, while Soxhlet extraction favored the recovery of other polar constituents.

## 3. Discussion

The results obtained in this study highlight the strong influence of solvent properties on the extraction of compounds from grape bagasse. The higher extraction yields observed for PLE compared to SFE are consistent with the polarity of the solvent system employed. Although ethanol was used as a co-extractant in SFE to enhance the extraction of polar compounds, its effect is limited by the predominance of supercritical CO_2_ in the solvent mixture, which restricts the overall polarity of the system [16]. In contrast, PLE employs ethanol as the main solvent under pressurized conditions, providing a more suitable environment for the solubilization of phenolic compounds [29,30]. This behavior is consistent with the composition of grape bagasse, in which phenolic compounds are mainly associated with the skins and seeds and are more effectively recovered using polar solvents [3,10].

Temperature also played a relevant role in the extraction process. For both PLE and SWE, increasing temperature led to higher extraction yields. In PLE, temperature showed a significant positive effect on extraction yield but did not significantly influence TPC. In SWE, increasing temperature promoted higher extraction yields as well as increases in phenolic content and antioxidant activity. However, the increase in extraction yield was more pronounced than that observed for PLE, while comparable TPC and antioxidant activity values were maintained between both techniques. These results suggest that elevated SWE temperatures favor not only the extraction of phenolic compounds, but also the recovery of additional highly polar non-phenolic constituents. In contrast, anthocyanin content decreased at higher temperatures, reflecting the instability of these compounds under more severe thermal conditions, as reported by Yammine et al. (2020), Castañeda-Ovando et al. (2009) and Patras et al. (2010) [24,31,32]. Similar effects of temperature on mass transfer and selectivity have been reported for plant matrices, where higher temperatures reduce solvent viscosity and increase diffusivity, but may also promote the co-extraction of non-target compounds [20,23,33,34].

This behavior highlights the trade-off between extraction yield and selectivity. While water-based extractions (Soxhlet and SWE) resulted in higher overall yields, they showed limited selectivity toward phenolic compounds. The increase in yield observed under these conditions is likely associated with the extraction of highly polar compounds, including soluble sugars and other low-molecular-weight constituents present in grape bagasse [10,35,36]. This interpretation is also supported by the proximate composition of the grape bagasse used in this study, which showed a predominance of carbohydrates and dietary fiber, components that may contribute directly or indirectly to the recovery of polar constituents during aqueous extraction processes. Although the present study focused on global parameters related to phenolic recovery and antioxidant activity, further chromatographic characterization could provide more detailed information regarding the composition of the extracts and the compounds co-extracted during the process. In contrast, the use of ethanol or ethanol–water mixtures provided a better balance between yield and phenolic recovery, reinforcing the importance of solvent polarity in defining extraction selectivity [12,37,38,39].

In addition to solvent composition, the liquid-to-solid ratio also influenced phenolic recovery during PLE. The significant quadratic effect observed for this variable indicates that both insufficient and excessive solvent volumes may limit extraction efficiency, demonstrating the importance of an appropriate balance between solvent availability and extract dilution for maximizing phenolic recovery. According to Rodrigues et al. (2023) [12], phenolic extraction is favored at intermediate liquid-to-solid ratios, close to 24 mL/g. Very low L/S ratios may impair extraction due to premature solvent saturation, limited compound diffusion, and increased mixture viscosity, whereas excessively high L/S ratios may promote excessive dilution of the extracted compounds, reducing the extraction driving force and increasing operational costs.

The influence of extraction selectivity was further evidenced by the sequential extraction strategies, which promoted the fractionation of compounds according to their polarity. In both low- and high-pressure approaches, the first extraction step, performed under conditions favoring phenolic recovery, accounted for most of the phenolic compounds and antioxidant activity. The second step, particularly when using water, primarily contributed to an increase in overall extraction yield, with minor effects on TPC and antioxidant activity. This pattern indicates that phenolic compounds were preferentially recovered in the first stage, while more polar constituents were extracted in subsequent steps. This behavior is consistent with the known influence of solvent properties on extraction selectivity, as reported for lignocellulosic biomasses [23,30,40,41]. From a practical perspective, this observation suggests that extraction processes targeting phenolic compounds may not require exhaustive extraction steps to achieve high recoveries. Since most phenolic compounds were recovered during the first extraction stage, the second step primarily contributed to biomass fractionation and overall yield rather than to the recovery of bioactive phenolics. This finding may be relevant for process optimization, as it indicates that extraction time, solvent consumption, and energy demand could potentially be reduced when the primary objective is the recovery of phenolic-rich fractions. Conversely, the subsequent aqueous extraction step may be advantageous when a broader utilization of the biomass is desired, enabling the recovery of additional polar constituents within a biorefinery approach.

In addition, the high-pressure sequential route showed greater selectivity toward phenolic compounds than the low-pressure Soxhlet-based route. Although the Soxhlet sequence resulted in higher overall extraction yield, the PLE–SWE sequence provided higher overall TPC, TAC, and antioxidant activity, indicating a more selective recovery of bioactive phenolic compounds. This behavior can be attributed to the enhanced mass transfer and solvent penetration promoted under pressurized conditions, which improve the accessibility of phenolic compounds within the lignocellulosic matrix while reducing the extensive co-extraction of other highly polar constituents commonly observed in prolonged Soxhlet extractions with water [13,42,43].

From a broader perspective, these findings indicate that extraction strategies can be tailored according to the target compounds and desired outcomes. Conditions that favor intermediate solvent polarity are more suitable for the selective recovery of phenolic compounds, whereas more polar systems promote higher overall yields due to the extraction of additional constituents. In this context, sequential extraction represents a viable strategy for the fractionation and valorization of grape bagasse, allowing the recovery of different classes of compounds within a single processing scheme, in line with the biorefinery concept [9,44,45].

Nevertheless, the industrial implementation of pressurized extraction technologies still depends on operational and economic factors, including equipment cost, energy demand, solvent consumption, and process integration. Since these aspects were beyond the scope of the present study, future investigations should include techno-economic and sustainability assessments to support industrial implementation. Therefore, the selection of extraction strategies should consider not only extraction efficiency and selectivity, but also scalability and process feasibility for large-scale applications.

The present study also has some limitations that should be acknowledged. The evaluation of extraction performance was based on global parameters, including total phenolic content, total anthocyanin content, and antioxidant activity, which are suitable for comparing the overall performance of the extraction strategies but do not allow the identification and quantification of individual compounds. Therefore, although the results indicate differences in extraction selectivity among the evaluated techniques, chromatographic analyses such as HPLC-DAD or LC-MS would provide a more comprehensive characterization of the extracts and a deeper understanding of the specific phenolic compounds recovered under each extraction condition. Furthermore, the sequential extraction strategies evaluated in this study were designed to maximize the recovery and fractionation of phenolic compounds and did not include an initial defatting step. From a biorefinery perspective, future studies could investigate the incorporation of a preliminary non-polar extraction stage to recover lipophilic compounds prior to the hydroethanolic and aqueous extraction steps, enabling a more comprehensive valorization of grape bagasse.

Furthermore, the experiments were performed using grape bagasse obtained from a single grape variety and source, and therefore the results should be interpreted within this specific context. Additional studies involving different grape varieties and cultivation conditions would contribute to evaluating the broader applicability of the proposed extraction strategies. Economic feasibility, energy consumption, and scale-up aspects were beyond the scope of the present study and should also be investigated in future work.

Finally, the results reported here are based on a single grape variety obtained from a single source. Although this approach ensured experimental consistency, further studies involving different grape cultivars and production regions would contribute to assessing the broader applicability of the proposed extraction strategies.

## 4. Materials and Methods

### 4.1. Raw Material and Centesimal Composition

Grape bagasse, consisting predominantly of skins, seeds, and residual pulp, with the presence of small amounts of stems, was obtained as a donation from the winery “Vô Vito”, located in São José dos Pinhais, PR, Brazil. The raw material originated from Bordô grapes (*Vitis labrusca*) cultivated in the Serra Gaúcha region, specifically in the Fazenda Souza district, Caxias do Sul, RS, Brazil.

The grape bagasse was dried in a tray dryer with forced air circulation (Fabbe, São Paulo, Brazil) at 60 °C for 24 h until reaching a moisture content below 5%. The dried material was then ground using a commercial blender. The samples were vacuum-packed in polyethylene bags and stored at −18 °C until further use.

Grape bagasse samples were characterized by AOAC methods [46,47,48], with moisture content of 4.54 ± 0.07% (method 926.12) [46] and total protein content of 11.28 ± 1.66% (method 928.08), for nitrogen-protein correspondence factor of 6.25 [47]. The ash content was 7.17 ± 0.45% (method 900.02) [47], fat content of 4.91 ± 0.29% (method 920.39 C) [48] and carbohydrates content of 72.10 ± 2.47%, obtained by difference.

### 4.2. Chemicals and Reagents

The analytical standards and reagents used were Folin–Ciocalteu, gallic acid, Trolox (6-hydroxy-2,5,7,8-tetramethylchroman-2-carboxylic acid), and ABTS (2,2′-azinobis-(3-ethylbenzothiazoline-6-sulfonic acid)), purchased from Sigma-Aldrich (Steinheim, Germany). Sodium carbonate, potassium persulfate, potassium chloride, and sodium acetate were of analytical grade. CO_2_ (99.9% purity, White Martins Ltd., Rio de Janeiro, Brazil), ethanol P.A. (Neon, São Paulo, Brazil), and distilled water were used in the preparation of the solutions.

### 4.3. Soxhlet Extractions

Soxhlet extraction was performed as a conventional method to obtain reference data. Briefly, 5 g of dried grape bagasse were extracted using 150 mL of solvent in a Soxhlet apparatus. The extraction was carried out for 6 h using different solvents, including ethanol and ethanol–water mixtures (50% and 70%, *v*/*v*), as well as water. Hydroethanolic mixtures were selected based on previous studies demonstrating their suitability for the extraction of phenolic compounds from grape pomace, providing an appropriate balance between solvent polarity and phenolic solubilization [12,37,38]. All experiments were performed in triplicate.

After extraction, ethanol was removed under reduced pressure using a rotary evaporator (Fisaton 802, São Paulo, Brazil). Aqueous extracts were subsequently freeze-dried (Liotop L 101, São Carlos, Brazil) to obtain moisture-free extracts. All extracts were stored at −18 °C until further analysis.

The extraction yield (%) was calculated based on the mass of crude extract obtained (m_extract_) and the initial mass of dry sample used in the extraction (m_sample_), according to Equation (1).

(1) $$\mathrm{Yield}\;\left(\%\right)\;=\;\frac{m_{\mathrm{extract}}}{m_{\mathrm{sample}}}\;\times \;100$$

### 4.4. Supercritical and Pressurized Liquid Extractions (Box–Behnken Design)

#### 4.4.1. Experimental Design

A Box–Behnken design (BBD) was employed to evaluate the effects of process variables on extraction performance for both supercritical fluid extraction (SFE) and pressurized liquid extraction (PLE). In each case, three independent variables were investigated at three levels (−1, 0, and +1).

For SFE, the evaluated variables were temperature (T, °C), pressure (P, bar), and the ethanol-to-solid ratio (E/S, g/g). For PLE, the variables included temperature (T, °C), ethanol concentration (EtOH, % *v*/*v*), and the liquid-to-solid ratio (L/S, mL/g). The experimental design consisted of 15 runs, including one central point performed in triplicate.

For SFE, the coded variables were defined as x_1_ (temperature), x_2_ (pressure), and x_3_ (ethanol-to-solid ratio). For PLE, the coded variables were defined as x_1_ (temperature), x_2_ (ethanol concentration), and x_3_ (liquid-to-solid ratio). The coding of the independent variables was performed according to Equation (2), where x_i_; is the coded value, X_i_; is the real value of the variable, X_0_ is the value at the central point, and ΔX represents the step size (variation between coded levels).

(2) $$x_{i}\;=\;\frac{X_{i}\;-\;X_{0}}{∆X}$$

The experimental data were fitted to second-order polynomial models, and the significance of the model and its terms was evaluated by analysis of variance (ANOVA). All statistical analyses were performed using Statistica 12.0 software (StatSoft Inc., Tulsa, OK, USA).

Model adequacy was assessed based on the coefficient of determination (R^2^) and the lack-of-fit test. The coefficient of determination (R^2^) indicates the proportion of the experimental variability explained by the model, with higher values reflecting a better fit between predicted and experimental data. The lack-of-fit test evaluates whether the residual variation can be attributed to random experimental error; therefore, non-significant lack-of-fit values (*p* > 0.05) indicate that the model adequately describes the experimental data within the studied experimental domain.

#### 4.4.2. Supercritical Fluid Extraction (SFE)

Supercritical fluid extraction (SFE) was performed using carbon dioxide (CO_2_) as the main solvent and ethanol as co-extractant, as described by Batista et al. (2025) [49] with some modifications. The experiments were conducted in a laboratory-scale stainless steel high-pressure extractor (22 cm length and 1.9 cm internal diameter), pressurized using a high-pressure syringe pump (ISCO, model 500 D, Lincoln, NE, USA). The extraction vessel was connected to a thermostatic bath (Nova Ética, model 521–5D, Vargem Grande Paulista, SP, Brazil) to control the operating temperature.

The effects of temperature (T, 40, 60, and 80 °C), pressure (P, 100, 175, and 250 bar), and ethanol-to-solid ratio (E/S, 0.5:1, 1.25:1, and 2:1 g/g) on the extraction performance were evaluated according to the Box–Behnken design described in Section 4.4.1. The SFE experimental ranges were selected based on previous studies involving the extraction of bioactive compounds from grape pomace and plant matrices using supercritical CO_2_ modified with ethanol as co-solvent, aiming to improve the recovery of moderately polar phenolic compounds while maintaining extraction selectivity [15,16,26,49].

The extraction procedure consisted of an initial static period of 30 min, followed by a dynamic extraction stage of 20 min. The solvent was delivered at a flow rate of 2 mL·min^−1^ through the grape bagasse bed.

The extracts were collected in pre-weighed test tubes and subsequently dried for yield determination (Equation (1)). All extracts were stored in amber vials at −18 °C until further analysis.

#### 4.4.3. Pressurized Liquid Extraction (PLE)

Pressurized liquid extraction (PLE) was performed on a self-assembled apparatus described by Gonçalves Rodrigues et al. (2019) [50] using ethanol and ethanol–water mixtures as extraction solvent. The experiments were carried out in a stainless-steel extraction cell (25 mm internal diameter and 180 mm height). The solvent was delivered using an HPLC pump (Waters, model 515, Milford, MA, USA), and the system pressure was maintained at 10 MPa using a needle valve (HiP, model 20–11LF4, Erie, PA, USA).

The effects of temperature (T, 40, 60, and 80 °C), ethanol concentration (EtOH, 50, 70, and 100% *v*/*v*), and liquid-to-solid ratio (L/S, 20, 26.67, and 33.33 mL/g) on the extraction performance were evaluated according to the Box–Behnken design described in Section 4.4.1. The liquid-to-solid ratio was controlled by adjusting the solvent flow rate. The selected temperature, ethanol concentration, and liquid-to-solid ratio ranges were based on studies reporting the recovery of phenolic compounds from grape pomace and plant matrices under pressurized conditions. The selected conditions aimed to evaluate the balance between extraction yield and phenolic selectivity while avoiding excessive thermal degradation of anthocyanins [29,30].

The experiments were performed in continuous mode for 20 min. In each run, 3 g of grape bagasse were mixed with approximately 60 g of glass beads and loaded into the extraction cell to ensure proper solvent distribution and prevent channeling.

The extracts obtained were subjected to the same post-processing procedure described for Soxhlet extraction, including solvent removal by rotary evaporation (for ethanol), followed by freeze-drying to remove water. The extraction yield was calculated according to Equation (1), and the dried extracts were stored at −18 °C until further analysis.

It should be noted that the extraction techniques evaluated in this study operate under different solvent delivery mechanisms and process configurations. In SFE, ethanol was used as a confined co-extractant in combination with flowing supercritical CO_2_, whereas in PLE the extraction solvent continuously flowed through the extraction cell. Consequently, the liquid-to-solid ratio is not directly comparable among the different extraction techniques, and the selected conditions were constrained by both the operational characteristics of each process and equipment limitations. Therefore, the results should be interpreted as a comparison of extraction performance among different technologies rather than as a direct assessment of solvent-to-solid ratio effects across methods.

### 4.5. Subcritical Water Extraction (SWE)

Subcritical water extraction (SWE) was performed using distilled water as extraction solvent, employing the same experimental apparatus and bed configuration described for PLE (3 g of grape bagasse mixed with approximately 60 g of glass beads) (Section 4.4.3).

The experiments were carried out at three temperatures (80, 100, and 120 °C), using the central flow rate and liquid-to-solid ratio (L/S) of the PLE experimental design (4 mL·min^−1^ and 26.67 mL/g, respectively). The extraction was performed in continuous mode for 20 min. The temperatures were selected according to previous studies on pressurized hot water extraction of phenolic compounds from winery by-products and plant matrices, considering the effect of temperature on water polarity, mass transfer, and extraction selectivity [20,23,33].

Extracts obtained were subjected to freeze-drying to remove water. The extraction yield was calculated according to Equation (1), and the dried extracts were stored at −18 °C until further analysis. All experiments were performed in duplicate.

### 4.6. Sequential Extractions

Sequential extractions were performed to promote the fractionation of soluble compounds present in grape bagasse by recovering distinct extract fractions. The procedure consisted of an initial extraction step using a hydroethanolic solvent, followed by a second step employing pure water. After the first extraction step, the residual biomass was dried in an oven at 60 °C for 24 h to remove residual solvent before being subjected to the subsequent extraction stage.

Two extraction routes were evaluated using this solvent sequence: a low-pressure approach based on Soxhlet extraction and a high-pressure approach combining pressurized liquid extraction (PLE) and subcritical water extraction (SWE).

The operating conditions for the first extraction step in both routes were defined based on the highest total phenolic content (TPC) obtained in the respective single-step experiments, corresponding to 70% ethanol for Soxhlet and PLE at 40 °C using 50% ethanol and a liquid-to-solid ratio (L/S) of 26.67 mL/g.

In the high-pressure route, the second step (SWE) was conducted at 120 °C (maintaining the same L/S), a condition previously associated with the highest extraction yield in SWE experiments.

The extracts obtained were subjected to freeze-drying to remove water. The extraction yield was calculated according to Equation (1) and the dried extracts were stored at −18 °C until further analysis.

### 4.7. Chemical Characterization

The chemical characterization of the extracts was performed by determining total phenolic content (TPC), total anthocyanin content (TAC), and antioxidant activity (ABTS assay). All analyses were carried out in triplicate.

Stock solutions were prepared by dissolving 10 mg of each extract in 1 mL of the same solvent used during the corresponding extraction procedure. The solutions were stored at −18 °C throughout the analytical period. When necessary, additional dilutions were performed to ensure that the absorbance values fell within the calibration ranges of the respective analytical methods.

#### 4.7.1. Total Phenolic Content (TPC)

The total phenolic content (TPC) was determined using the Folin–Ciocalteu method, according to Singleton et al. (1999) [51], with minor adaptations. Briefly, 10 μL of appropriately diluted extract were mixed with 600 μL of distilled water and 50 μL of Folin–Ciocalteu reagent. After 1 min, 150 μL of 20% (*w*/*v*) sodium carbonate solution were added, followed by 190 μL of distilled water. The mixture was vortexed and kept in the dark for 2 h.

An aliquot of 300 μL was transferred to a microplate (in triplicate), and the absorbance was measured at 760 nm using a spectrophotometer (Agilent, Epoch-BioTek, Winooski, VT, USA).

Quantification was performed using a gallic acid calibration curve, and the results were expressed as mg of gallic acid equivalents per gram of dry extract (mg GAE g^−1^).

#### 4.7.2. Total Anthocyanin Content (TAC)

The total anthocyanin content (TAC) was determined by the pH differential method, as described by Giusti and Wrolstad (2001) [52]. Extracts were diluted in buffer solutions at pH 1.0 (potassium chloride, 0.025 mol/L) and pH 4.5 (sodium acetate, 0.4 mol/L).

Aliquots of 20 μL of extract were mixed with 280 μL of each buffer solution in a microplate (in triplicate). Absorbance was measured at 520 and 700 nm using a spectrophotometer (Agilent, Epoch-BioTek, Winooski, VT, USA).

The total monomeric anthocyanin content was calculated and expressed as mg of cyanidin-3-O-glucoside equivalents per gram of dry extract (mg CY g^−1^).

#### 4.7.3. Antioxidant Activity (ABTS Assay)

The antioxidant activity was determined using the ABTS radical cation decolorization assay, according to Re et al. (1999) [53]. The ABTS•^+^ radical was generated by reacting ABTS (7 mmol/L) with potassium persulfate (2.45 mmol/L) and allowing the mixture to stand in the dark at room temperature for 16 h.

The solution was diluted with distilled water to obtain an absorbance of 0.70 ± 0.02 at 734 nm. For the assay, 20 μL of diluted extract were added to 280 μL of ABTS solution in a microplate (in triplicate). After 30 min in the dark, the absorbance was measured at 734 nm (Agilent, Epoch-BioTek, Winooski, VT, USA).

The results were expressed as μmol of Trolox equivalents per gram of dry extract (μmol TE g^−1^), based on a Trolox calibration curve.

## 5. Conclusions

The extraction technique and solvent system strongly influenced the recovery of bioactive compounds from grape bagasse. Hydroethanolic extractions showed greater selectivity toward phenolic compounds, while water-based extractions provided higher extraction yields associated with the recovery of additional highly polar compounds. Among the pressurized techniques, PLE resulted in higher extraction yields than SFE, whereas SWE promoted higher extraction yields with comparable TPC and antioxidant activity values relative to PLE, which may be associated with the co-extraction of additional polar constituents beyond phenolic compounds. Higher SWE temperatures also led to lower anthocyanin contents, demonstrating the susceptibility of these compounds to elevated temperatures.

Sequential extraction strategies demonstrated the potential for fractionating grape bagasse components according to their polarity. In both low- and high-pressure routes, the first extraction step accounted for most of the phenolic recovery and antioxidant activity, while the second aqueous step mainly increased the overall extraction yield. The sequential PLE–SWE approach resulted in the highest overall TPC and antioxidant activity, highlighting its potential for grape bagasse valorization within a biorefinery context.

These findings demonstrate that the selection and combination of extraction technologies can be tailored according to the desired product profile, enabling the selective recovery of bioactive compounds while maximizing biomass utilization. By systematically comparing conventional, supercritical, and pressurized extraction technologies, as well as their application in sequential fractionation strategies, this study provides insights into the relationship between extraction conditions, selectivity, and phenolic recovery from grape bagasse. The superior performance of the PLE–SWE sequence highlights the potential of integrating pressurized extraction technologies for biomass valorization within a biorefinery framework. Although the PLE–SWE strategy showed the most promising performance for phenolic recovery, future studies should evaluate its economic viability, energy requirements, solvent consumption, and scalability in order to support industrial implementation.

## Figures and Tables

**Table 1:** Extraction yield, total phenolic content (TPC), total anthocyanin content (TAC), and antioxidant activity (ABTS) of Soxhlet extracts obtained using different ethanol concentrations.

EtOH (% *v*/*v*)	Yield (%)	TPC (mg GAE g^−1^)	TAC (mg CY g^−1^)	ABTS (μmol TE g^−1^)
100	14.0 ± 0.7	52.7 ± 0.8	5.7 ± 0.2	667 ± 14
70	17.8 ± 0.8	100.3 ± 0.9	11.5 ± 0.8	1358 ± 31
50	17.5 ± 0.2	98.2 ± 0.1	6.4 ± 0.2	1447 ± 22
0	26.1 ± 0.8	54.6 ± 0.6	3.7 ± 0.1	916 ± 25

**Table 2:** Predictive models obtained from the Box–Behnken experimental designs for supercritical fluid extraction (SFE) and pressurized liquid extraction (PLE), including extraction yield, total phenolic content (TPC), total anthocyanin content (TAC), and antioxidant activity (ABTS). Coefficients of determination (R^2^) and lack-of-fit *p*-values are also presented. The models are expressed in terms of coded variables. For SFE, x_1_ = temperature, x_2_ = pressure, and x_3_ = ethanol-to-solid ratio. For PLE, x_1_ = temperature, x_2_ = ethanol concentration, and x_3_ = liquid-to-solid ratio.

Method	Response	Model	R^2^	Lack of Fit *p*-Value
SFE	Yield (%) =	$6.32+1.74\left(x_{1}\right)\;+1.27\left(x_{3}\right)$	0.98	0.2474
PLE	Yield (%) =	$14.92+2.08\left(x_{1}\right)-1.18\left(x_{2}\right)-0.74{\left(x_{1}\right)}^{2}$	0.92	0.1604
	TPC (mg GAE g^−1^) =	$84.97-30.92\left(x_{2}\right)+9.35{\left(x_{2}\right)}^{2}+8.85{\left(x_{3}\right)}^{2}-14.46\left(x_{1}\right)\left(x_{3}\right)$	0.94	0.1357
	TAC (mg CY g^−1^) =	$15.67-5.21\left(x_{2}\right)+3.59{\left(x_{2}\right)}^{2}+5.50\left(x_{1}\right)\left(x_{2}\right)+3.71\left(x_{2}\right)\left(x_{3}\right)$	0.89	0.0794
	ABTS (μmol TE g^−1^) =	$857.00+190.65\left(x_{1}\right)-254.93\left(x_{2}\right)+167.56{\left(x_{2}\right)}^{2}+126.85{\left(x_{3}\right)}^{2}$	0.88	0.1338

**Table 3:** Extraction yield, total phenolic content (TPC), total anthocyanin content (TAC), and antioxidant activity (ABTS) of extracts obtained by subcritical water extraction (SWE) at different temperatures.

Temperature (°C)	Yield (%)	TPC (mg GAE g^−1^)	TAC (mg CY g^−1^)	ABTS (μmol TE g^−1^)
80	22.6 ± 1.3	53.9 ± 0.9	10.3 ± 2.0	731 ± 31
100	24.5 ± 0.2	76.6 ± 0.2	8.9 ± 1.5	887 ± 7
120	26.7 ± 0.8	103.9 ± 1.0	5.8 ± 1.3	1125 ± 24

**Table 4:** Extraction yield, total phenolic content (TPC), total anthocyanin content (TAC), and antioxidant activity (ABTS) obtained from sequential extraction strategies using low-pressure (↓P) and high-pressure (↑P) extraction routes applied to grape bagasse.

	Method	Yield (%)	TPC (mg GAE g^−1^)	TAC (mg CY g^−1^)	ABTS (μmol TE g^−1^)
↓P	(1º) SOX (EtOH 70%)	17.8 ± 0.8	100.3 ± 0.9	11.5 ± 0.8	1358 ± 31
	(2º) SOX (Water)	24.2 ± 1.7	45.3 ± 0.1	0.2 ± 0.1	891 ± 21
	TOTAL:	42.0 ± 1.9	145.6 ± 1.0	11.7 ± 0.8	2249 ± 37
↑P	(1º) PLE (40 °C; EtOH 50%; L/S 26.67 mL/g)	13.5 ± 0.8	129.4 ± 0.6	23.0 ± 0.8	1304 ± 22
	(2º) SWE (120 °C)	18.4 ± 0.3	68.6 ± 0.2	1.8 ± 0.3	1017 ± 14
	TOTAL:	31.9 ± 0.9	198.0 ± 0.7	24.8 ± 0.9	2321 ± 26

## Data Availability

The data supporting the findings of this study are available within the article and its Supplementary Materials. Additional data are available from the corresponding author upon reasonable request.

## Supplementary Materials

The following supporting information can be downloaded at: https://www.mdpi.com/article/10.3390/molecules31132314/s1, Figure S1: Extraction kinetics fitted by the PieceWise model in Origin software for the different extraction methods: (a) Supercritical Fluid Extraction (SFE), (b) Pressurized Liquid Extraction (PLE), and (c) Subcritical Water Extraction (SWE). The PieceWise model divides the extraction process into three distinct phases: Constant Extraction Rate (CER), characterized by rapid extraction controlled by solute convection from the particle surface; Falling Extraction Rate (FER), associated with a gradual decrease in mass transfer due to partial depletion of readily accessible solutes; and Diffusion-Controlled Rate (DCR), in which extraction is governed by the diffusion of remaining solutes from the solid matrix interior; Table S1: Experimental responses of extraction yield obtained from the Box–Behnken Design (BBD) applied to the Supercritical Fluid Extraction (SFE) method; Table S2: Experimental responses of extraction yield, total phenolic content (TPC), total anthocyanin content (TAC), and antioxidant activity determined by the ABTS assay obtained from the Box–Behnken Design (BBD) applied to the Pressurized Liquid Extraction (PLE) method; Table S3: ANOVA for the quadratic model fitted to extraction yield obtained from the Box–Behnken Design (BBD) for the SFE process; Table S4: ANOVA for the quadratic model fitted to extraction yield obtained from the Box–Behnken Design (BBD) for the PLE process; Table S5: ANOVA for the quadratic model fitted to total phenolic content (TPC) obtained from the Box–Behnken Design (BBD) for the PLE process; Table S6: ANOVA for the quadratic model fitted to total anthocyanin content (TAC) obtained from the Box–Behnken Design (BBD) for the PLE process; Table S7: ANOVA for the quadratic model fitted to antioxidant activity (ABTS) obtained from the Box–Behnken Design (BBD) for the PLE process. Figure S2: Response surface plots obtained from the Box–Behnken Design (BBD) for the extraction yield response of the Supercritical Fluid Extraction (SFE) process: (a) effect of pressure and temperature; (b) effect of ethanol-to-solid ratio (E/S) and temperature; and (c) effect of ethanol-to-solid ratio (E/S) and pressure; Figure S3: Response surface plots obtained from the Box–Behnken Design (BBD) for the extraction yield response of the Pressurized Liquid Extraction (PLE) process: (a) effect of ethanol concentration (EtOH%) and temperature; (b) effect of liquid-to-solid ratio (L/S) and temperature; and (c) effect of liquid-to-solid ratio (L/S) and ethanol concentration (EtOH%); Figure S4: Response surface plots obtained from the Box–Behnken Design (BBD) for the total phenolic content (TPC) response of the Pressurized Liquid Extraction (PLE) process: (a) effect of ethanol concentration (EtOH%) and temperature; (b) effect of liquid-to-solid ratio (L/S) and temperature; and (c) effect of liquid-to-solid ratio (L/S) and ethanol concentration EtOH (% *v*/*v*); Figure S5: Response surface plots obtained from the Box–Behnken Design (BBD) for the total anthocyanin content (TAC) response of the Pressurized Liquid Extraction (PLE) process: (a) effect of ethanol concentration (EtOH%) and temperature; (b) effect of liquid-to-solid ratio (L/S) and temperature; and (c) effect of liquid-to-solid ratio (L/S) and ethanol concentration (EtOH%); Figure S6: Response surface plots obtained from the Box–Behnken Design (BBD) for the antioxidant activity (ABTS) response of the Pressurized Liquid Extraction (PLE) process: (a) effect of ethanol concentration (EtOH%) and temperature; (b) effect of liquid-to-solid ratio (L/S) and temperature; and (c) effect of liquid-to-solid ratio (L/S) and ethanol concentration (EtOH%).

## Abbreviations

The following abbreviations are used in this manuscript:

ABTS	2,2′-azinobis-(3-ethylbenzothiazoline-6-sulfonic acid)
BBD	Box–Behnken design
CY	Cyanidin equivalents
GAE	Gallic acid equivalents
PLE	Pressurized liquid extraction
SFE	Supercritical fluid extraction
SWE	Subcritical water extraction
TAC	Total anthocyanin content
TE	Trolox equivalents
TPC	Total phenolic content
