# Supplementary material for: Sequential Pressurized and Supercritical Extraction Strategies for the Recovery of Phenolic Compounds and Grape Bagasse Valorization
Source: Molecules. 2026 Jul 1;31(13):2314. doi: 10.3390/molecules31132314 (PMC13362762; doi:10.3390/molecules31132314)
Supplement: Supplementary file 1 [file molecules-31-02314-s001.zip › molecules-4358104-supplementary.pdf]

Supplementary Materials

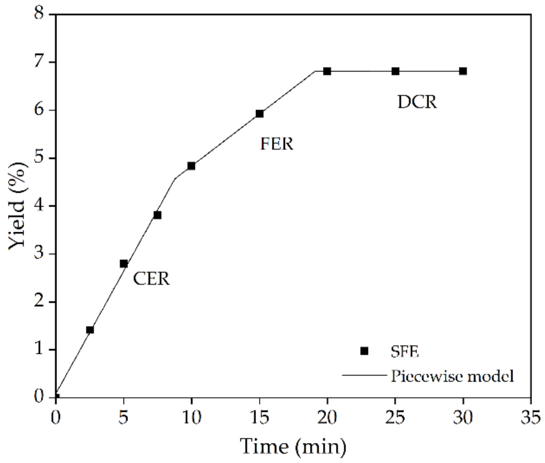

(**a**)

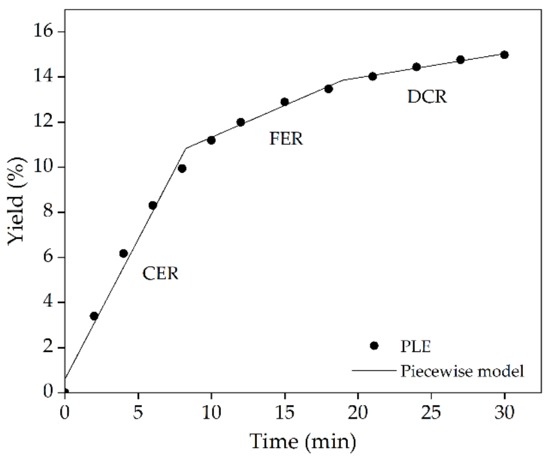

(**b**)

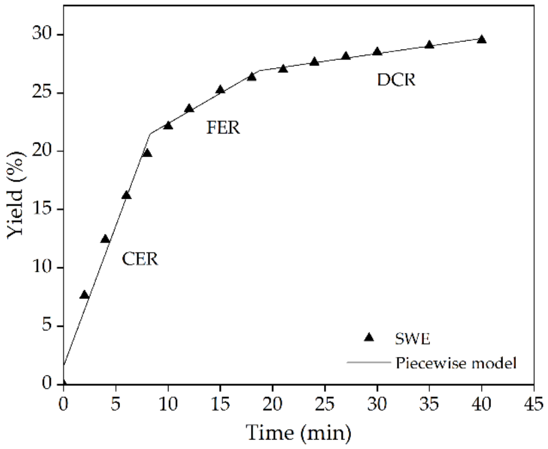

(**c**)

Figure S1. Extraction kinetics fitted by the PieceWise model in Origin software for the different extraction methods: (**a**) Supercritical Fluid Extraction (SFE), (**b**) Pressurized Liquid Extraction (PLE), and (**c**) Subcritical Water Extraction (SWE). The PieceWise model divides the extraction process into three distinct phases: Constant Extraction Rate (CER), characterized by rapid extraction controlled by solute convection from the particle surface; Falling Extraction Rate (FER), associated with a gradual decrease in mass transfer due to partial depletion of readily accessible solutes; and Diffusion-Controlled Rate (DCR), in which extraction is governed by the diffusion of remaining solutes from the solid matrix interior.

Table S1. Experimental responses of extraction yield obtained from the Box–Behnken Design (BBD) applied to the Supercritical Fluid Extraction (SFE) method.

Conditions assessed			**Yield (%)**
Temperature (°C)	Pressure (bar)	E/S (g/g)	
40	100	1	5.19
80	100	1.25	7.53
40	250	1.25	4.75
80	250	1.25	8.20
40	175	0.5	3.54
80	175	0.5	7.02
40	175	2	5.42
80	175	2	10.10
60	100	0.5	4.26
60	250	0.5	5.16
60	100	2	7.28
60	250	2	7.36
60	175	1.25	7.25
60	175	1.25	6.82
60	175	1.25	6.71

Table S2. Experimental responses of extraction yield, total phenolic content (TPC), total anthocyanin content (TAC), and antioxidant activity determined by the ABTS assay obtained from the Box–Behnken Design (BBD) applied to the Pressurized Liquid Extraction (PLE) method.

Conditions assessed			**Yield (%)**	**TPC (mg GAE g^{-1})**	**TAC (mg CY g^{-1})**	**ABTS (umol TE g^{-1})**
Temperature (°C)	EtOH (% v/v)	L/S (mL g^{-1})				
40	50	26.67	13.2	129.0 ± 1.7	24.1 ± 0.9	1159 ± 60
80	50	26.67	18.0	110.7 ± 1.6	12.5 ± 0.4	1105 ± 23
40	100	26.67	11.9	56.8 ± 1.5	6.4 ± 0.8	519 ± 29
80	100	26.67	17.0	67.6 ± 0.3	16.9 ± 0.6	929 ± 45
40	70	20	15.5	68.5 ± 0.3	17.4 ± 0.8	623 ± 13
80	70	20	17.2	123.6 ± 0.9	18.5 ± 0.7	1112 ± 28
40	70	33.33	14.1	101.7 ± 2.2	25.8 ± 0.1	951 ± 33
80	70	33.33	19.0	99.0 ± 1.0	19.7 ± 1.3	1556 ± 5
60	50	20	14.6	120.7 ± 1.5	25.7 ± 0.4	981 ± 53
60	100	20	11.7	32.1 ± 1.1	3.2 ± 0.8	331 ± 18
60	50	33.33	16.1	89.0 ± 2.2	13.8 ± 0.0	898 ± 57
60	100	33.33	11.8	45.5 ± 1.1	8.1 ± 0.6	324 ± 9
60	70	26.67	15.4	120.6 ± 1.1	24.9 ± 0.0	1389 ± 42
60	70	26.67	14.4	114.3 ± 1.3	24.3 ± 0.3	1222 ± 48
60	70	26.67	15.1	108.5 ± 1.2	22.1 ± 0.5	1209 ± 63

Table S3. ANOVA for the quadratic model fitted to extraction yield obtained from the Box–Behnken Design (BBD) for the SFE process.

Factor	SS	df	MS	F	p
(1) Temperature (°C) (L)	24.32531	1	24.32531	298.7144	0.003331
Temperature (°C) (Q)	0.00002	1	0.00002	0.0002	0.990081
(2) Pressure (bar) (L)	0.18301	1	0.18301	2.2474	0.272592
Pressure (bar) (Q)	0.94942	1	0.94942	11.6588	0.076109
(3) E/S (g/g) (L)	12.95405	1	12.95405	159.0755	0.006228
E/S (g/g) (Q)	0.60439	1	0.60439	7.4218	0.112460
1L by 2L	0.30802	1	0.30802	3.7825	0.191216
1L by 3L	0.36000	1	0.36000	4.4208	0.170234
2L by 3L	0.16810	1	0.16810	2.0643	0.287325
Lack of Fit	0.78088	3	0.26029	3.1964	0.247351
Pure Error	0.16287	2	0.08143		
Total SS	40.70289	14			

Table S4. ANOVA for the quadratic model fitted to extraction yield obtained from the Box–Behnken Design (BBD) for the PLE process.

Factor	SS	df	MS	F	p
(1) Temperature (°C) (L)	33.82096	1	33.82096	112.9574	0.008737
Temperature (°C) (Q)	7.98664	1	7.98664	26.6743	0.035505
(2) EtOH (%) (L)	11.06110	1	11.06110	36.9426	0.026017
EtOH (%) (Q)	5.07966	1	5.07966	16.9654	0.054196
(3) L/S (mL/g) (L)	0.42352	1	0.42352	1.4145	0.356369
L/S (mL/g) (Q)	0.00159	1	0.00159	0.0053	0.948531
1L by 2L	0.11978	1	0.11978	0.4000	0.591731
1L by 3L	2.66550	1	2.66550	8.9024	0.096366
2L by 3L	0.38334	1	0.38334	1.2803	0.375260
Lack of Fit	4.84388	3	1.61463	5.3926	0.160409
Pure Error	0.59883	2	0.29941		
Total SS	70.86057	14			

Table S5. ANOVA for the quadratic model fitted to total phenolic content (TPC) obtained from the Box–Behnken Design (BBD) for the PLE process.

Factor	SS	df	MS	F	p
(1) Temperature (°C) (L)	300.90	1	300.895	8.1740	0.103663
Temperature (°C) (Q)	8.04	1	8.038	0.2184	0.686258
(2) EtOH (%) (L)	7647.21	1	7647.209	207.7408	0.004779
EtOH (%) (Q)	1268.23	1	1268.233	34.4523	0.027820
(3) L/S (mL/g) (L)	0.25	1	0.247	0.0067	0.942138
L/S (mL/g) (Q)	1157.70	1	1157.699	31.4496	0.030357
1L by 2L	131.29	1	131.289	3.5665	0.199556
1L by 3L	836.49	1	836.486	22.7236	0.041300
2L by 3L	442.32	1	442.319	12.0158	0.074093
Lack of Fit	720.85	3	240.283	6.5274	0.135731
Pure Error	73.62	2	36.811		
Total SS	13403.61	14			

Table S6. ANOVA for the quadratic model fitted to total anthocyanin content (TAC) obtained from the Box–Behnken Design (BBD) for the PLE process.

Factor	SS	df	MS	F	p
(1) Temperature (°C) (L)	0.3546	1	0.3546	0.16236	0.725986
Temperature (°C) (Q)	1.1670	1	1.1670	0.53431	0.540837
(2) EtOH (%) (L)	216.8314	1	216.8314	99.27570	0.009923
EtOH (%) (Q)	186.7911	1	186.7911	85.52183	0.011492
(3) L/S (mL/g) (L)	3.8770	1	3.8770	1.77507	0.314282
L/S (mL/g) (Q)	29.8052	1	29.8052	13.64622	0.066098
1L by 2L	123.6351	1	123.6351	56.60601	0.017211
1L by 3L	13.1051	1	13.1051	6.00013	0.133972
2L by 3L	56.2913	1	56.2913	25.77283	0.036679
Lack of Fit	77.0064	3	25.6688	11.75239	0.079431
Pure Error	4.3683	2	2.1841		
Total SS	764.4006	14			

Table S7. ANOVA for the quadratic model fitted to antioxidant activity (ABTS) obtained from the Box–Behnken Design (BBD) for the PLE process.

Factor	SS	df	MS	F	p
(1) Temperature (°C) (L)	285171	1	285171.2	28.46680	0.033380
Temperature (°C) (Q)	6089	1	6089.4	0.60787	0.517206
(2) EtOH (%) (L)	519931	1	519930.7	51.90132	0.018728
EtOH (%) (Q)	407132	1	407131.5	40.64131	0.023733
(3) L/S (mL/g) (L)	56634	1	56633.7	5.65338	0.140537
L/S (mL/g) (Q)	237633	1	237633.0	23.72136	0.039665
1L by 2L	37390	1	37389.9	3.73239	0.193089
1L by 3L	3360	1	3359.6	0.33537	0.621048
2L by 3L	28	1	27.8	0.00278	0.962771
Lack of Fit	199316	3	66438.8	6.63216	0.133831
Pure Error	20035	2	10017.7		
Total SS	1865188	14			

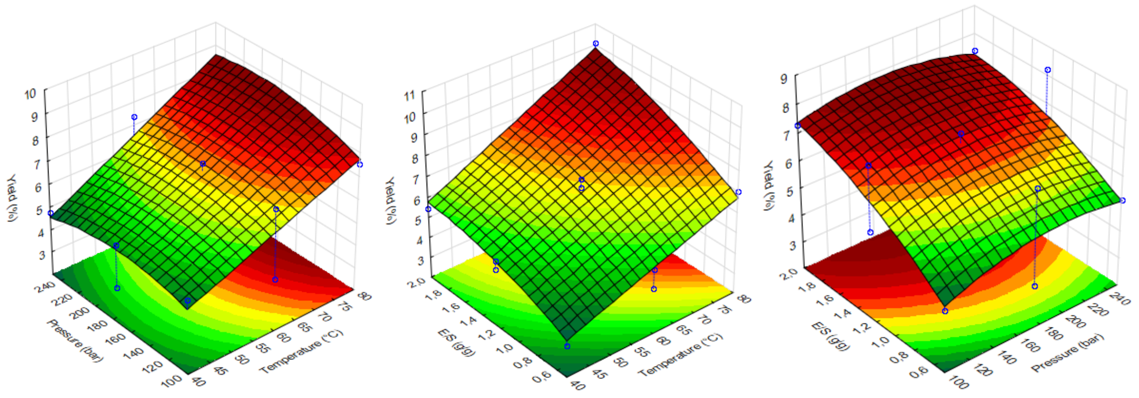

(**a**) (**b**) (**c**)

Figure S2. Response surface plots obtained from the Box–Behnken Design (BBD) for the extraction yield response of the Supercritical Fluid Extraction (SFE) process: (a) effect of pressure and temperature; (b) effect of ethanol-to-solid ratio (E/S) and temperature; and (c) effect of ethanol-to-solid ratio (E/S) and pressure.

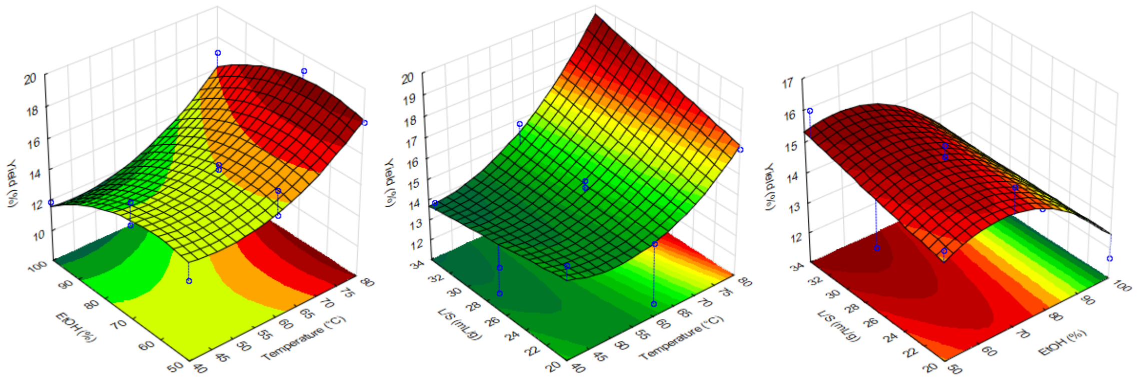

(**a**) (**b**) (**c**)

Figure S3. Response surface plots obtained from the Box–Behnken Design (BBD) for the extraction yield response of the Pressurized Liquid Extraction (PLE) process: (a) effect of ethanol concentration (EtOH%) and temperature; (b) effect of liquid-to-solid ratio (L/S) and temperature; and (c) effect of liquid-to-solid ratio (L/S) and ethanol concentration (EtOH%).

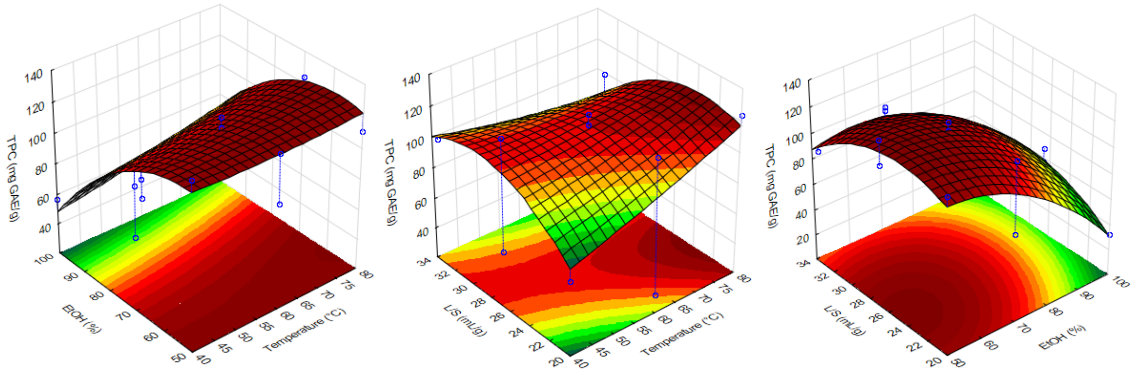

(**a**) (**b**) (**c**)

Figure S4. Response surface plots obtained from the Box–Behnken Design (BBD) for the total phenolic content (TPC) response of the Pressurized Liquid Extraction (PLE) process: (a) effect of ethanol concentration (EtOH%) and temperature; (b) effect of liquid-to-solid ratio (L/S) and temperature; and (c) effect of liquid-to-solid ratio (L/S) and ethanol concentration (EtOH%).

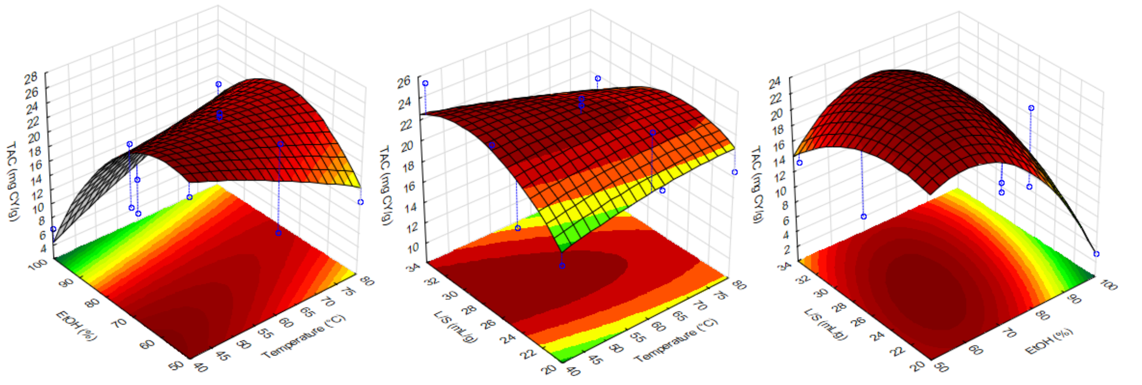

(**a**) (**b**) (**c**)

Figure S5. Response surface plots obtained from the Box–Behnken Design (BBD) for the total anthocyanin content (TAC) response of the Pressurized Liquid Extraction (PLE) process: (a) effect of ethanol concentration (EtOH%) and temperature; (b) effect of liquid-to-solid ratio (L/S) and temperature; and (c) effect of liquid-to-solid ratio (L/S) and ethanol concentration (EtOH%).

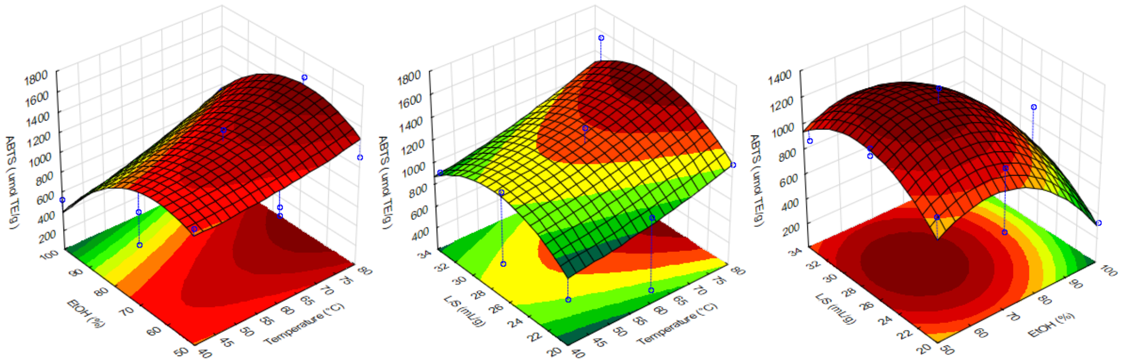

(**a**) (**b**) (**c**)

Figure S6. Response surface plots obtained from the Box–Behnken Design (BBD) for the antioxidant activity (ABTS) response of the Pressurized Liquid Extraction (PLE) process: (a) effect of ethanol concentration (EtOH%) and temperature; (b) effect of liquid-to-solid ratio (L/S) and temperature; and (c) effect of liquid-to-solid ratio (L/S) and ethanol concentration (EtOH%).
